# Dynamic analysis and optimal control of knowledge diffusion model in regional innovation ecosystem under digitalization

**DOI:** 10.1038/s41598-024-63634-3

**Published:** 2024-06-07

**Authors:** Zitong He, Haijun Wang, Yuhan Hu, Xiaolin Ma, Huiyan Zhao

**Affiliations:** 1 School of Management, Shenyang University of Technology, Shenyang, 110870 China; 2 School of Science, University of Science and Technology Liaoning, Anshan, 114051 China; 3 School of Business Administration, University of Science and Technology Liaoning, Anshan, 114051 China

**Keywords:** Applied mathematics, Environmental economics

## Abstract

Knowledge diffusion in regional innovation ecosystems is an important factor that influences the regional innovation efficiency. In regional innovation ecosystems under digital empowerment, the knowledge diffusion enables the optimal allocation of innovation resources and promotes the sustainable development and ecological evolution of regional innovation ecosystems. In this paper, a SEIR (Susceptible–Exposed–Infected–Recovered) model is proposed for knowledge diffusion in regional innovation ecosystems under digitization. The basic reproduction number of the proposed model is calculated and its stability is validated. Finally, the expressions for the optimal control system and the optimal control parameters are presented. According to the research conclusions of this paper, the knowledge-diffusion ability of innovation agents in an innovation ecosystem affects the knowledge diffusion in a system; the contact rate between innovation agents affects the efficiency of knowledge diffusion in the system and the structure of the system; the digital transmission ability of innovation agents affects the breadth of knowledge diffusion in the system; and the self-learning ability of innovation agents affects the efficiency of knowledge diffusion in the system.The digital technologies help heterogeneous innovation agents in regional innovation ecosystems to break down the knowledge silos. At the same time, the digital technologies enhance the ability of innovation agents to absorb and learn knowledge in regional innovation ecosystems under digitization, thereby increasing the infection rate of knowledge diffusion in such systems.These conclusions extend the theoretical boundaries of innovation ecosystems and knowledge diffusion and offer management implications for enterprises and governments in decision-making.

## Introduction

Innovation is a core driver in national social development. It plays a key role in enhancing the competitive advantages of organizations^1–3^ . In global technological advancements and rapid industrial evolution, scientific and technological innovation has become the foundation of national economic competitiveness^4^. Due to the development of economic globalization, the innovation competition between different regions has evolved into a competition between regional systems and ecological dimensions^5,6^ . Consequently, a series of regional innovation centers have emerged in different countries. In this context, improving regional innovation capabilities and designing scientific and efficient innovative development models plays crucial role in the current economic development in developed as well as in developing countries. Currently, regional innovative development model has evolved from a linear single model (innovation 1.0 stage) into an innovation system model (innovation 2.0 stage), and finally into an innovation ecosystem model (innovation 3.0 stage)^7,8^. Supported by the collaborative innovation, the regional innovation ecosystems aim at symbiosis and a win-win situation by attempting to achieve innovation resource sharing, complementary advantages, and risk sharing^9^ . It is noteworthy that the evolutionary process of regional innovation ecosystems is dynamic and complex^10,11^ . Huang^12^ described the characteristics of regional innovation ecosystems from an ecological perspective. Similar to the natural ecosystems, regional innovation ecosystems are integral, hierarchical, dissipative, dynamic, stable, complex, and regulable. As far as regional industrial development is concerned, knowledge resources constitute the first element of scientific and technological innovation. In fact, the role of technology and knowledge innovation in increasing the innovation production efficiency of systems has far exceeded the roles of capital and labor in traditional models. The knowledge innovation is not only the key in maintaining the core competitiveness of enterprises, but it is also a critical factor that drives the regional economic growth^13^ . Considering the perspective of knowledge flow in systems, knowledge absorption is an important pathway based on which the innovation agents learn^14^ . On the other hand, knowledge diffusion is an intermediate link that engages the innovation agents in knowledge innovation and application, and is a source of competitive advantage for systems in the dynamic evolution of innovation environments^15^ .

The book *DiffusionofInnovations*, authored by American scholar Everett M. Rogers in the 1960s is the first source to consider the treating individuals’ absorption of innovative ideas as an uncontrolled epidemic^16^. In innovation ecosystems, knowledge flow is often described as knowledge exchange, knowledge innovation, and knowledge infection (such as epidemic spreading models)^17,18^. It is noteworthy that the knowledge diffusion in ecosystems shows certain characteristics and patterns, which are similar to the spreading of infectious pathogens in populations. Therefore, the epidemic diffusion models can be employed to explore knowledge diffusion. There are few works presented in literature that have explored the evolutionary mechanism of knowledge diffusion and dissemination in innovation ecosystems by constructing knowledge diffusion models. Baggio et al. constructed a susceptible-infected (SI) epidemic model to investigate the online knowledge dissemination^19^. Based on the susceptible-infected-recovered (SIR) model, Zhang et al.^20^ proposed a knowledge diffusion model based on relational networks. Wang et al.^21^ constructed a knowledge diffusion model by considering the self-learning mechanisms based on the SIR model. Kabir et al.^22^ inquired the information dissemination using the SIR model. Based on the social network analysis and regression analysis, Ye et al.^23^ probed into the problem of community knowledge dissemination using the SIR model and discussed that the regional characteristics and innovation recognition of community subjects affect the scope and rate of knowledge dissemination. It can be seen that knowledge diffusion is essentially a behavioral process of knowledge dissemination in complex networks. Please note that the process of knowledge diffusion is complex and dynamic^24,25^ , while the epidemic models provide strong support for the dynamic process of knowledge diffusion in systems.

Knowledge diffusion in any regional innovation ecosystem refers to the diffusion and dissemination of cutting-edge technologies and innovative knowledge developed in a system through a complex regional innovation network, leading to the evolution of knowledge from the production stage to the transformation of scientific and technological achievements^26^. As an important concept in the theoretical system of regional innovation^27^ , knowledge diffusion is not only an important cause of regional innovation ecosystems, but it is also an important factor that influences their innovation efficiency^28^. Based on knowledge diffusion, regional innovation ecosystems achieve knowledge resources sharing and complementary advantages between innovation agents in different regions, thereby maximizing the utilization of knowledge resources under a certain stock^29^. The free flow of innovation agents inside and outside a regional innovation ecosystem promotes the diffusion and flow of knowledge. Furthermore, the application of knowledge by the heterogeneous innovation agents in the system drives knowledge transfer and diffusion between subjects in the system^30^ . The digital technologies have a positive promoting effect on knowledge diffusion, which in turn serves as a critical factor in promoting the productivity^31^. On one hand, the digitization lifts spatio-temporal restrictions and accelerates the storage and diffusion of knowledge to some extent^32^ . On the other hand, with the aid of heterogeneous digital platforms, the knowledge breaks through the spatial limitations and barriers, thus increasing the efficiency of enterprises in learning the external knowledge and experience, and expand the scope of knowledge spillover. With the deepening of digitization, it is possible for innovative subjects to acquire knowledge on a large scale. The knowledge flow formed by the diffusion of knowledge among individuals is a form of human communication. The diffusion of knowledge depends on the development of digital technology, and the development of diffusion means plays an irreplaceable role in promoting the evolution of digital media. For example, due to the backward digital technology in ancient society, information can only be transmitted by oral transmission and letters. After the emergence of digital technology, the emergence of new media such as network media and mobile phone media has promoted the change of the mode and pattern of knowledge diffusion, and thus profoundly promoted the social reform. Knowledge diffusion has presented a very good development prospect with the promotion of digital technology.

The digital regional innovation ecosystem diverges significantly traditional non-digital counterpart. The digital regional innovation ecosystem is distinguished by its interactive and symbiotic attributes, whereas the non-digital variant is characterized by independence and delay, with relatively independent innovation agents and a sluggish flow of resource flow. The digital regional innovation ecosystem effectively addresses these limitations. In the context of digitization, the regional innovation ecosystem utilizes digital technology and data elements to create new products and value , connecting the complex networks among participants across different tiers of the system in a digital manner . On the basis of the non-digital traditional regional innovation ecosystem, the digital regional innovation ecosystem markedly enhances the level of collaboration among innovation entities and facilitates the shared utilization of innovation resources .

The existing studies regarding knowledge diffusion mainly focus on two aspects, i.e., diffusion factors and channels. In terms of diffusion factors, Rogers discussed that the knowledge diffusion plays a vital role in social systems, information and knowledge channels, innovation, timing, etc. In terms of diffusion channels, Fourt, Woodlock, and Mansfield concentrated on the diffusion channels. Bass introduced an knowledge diffusion rate model and identified the mass media and word-of-mouth communication as the main channels of innovation diffusion^33^. The scholars, such as Harkola, Abrahamson, Goldenberg, and Garber conducted their research and analysis mainly using the real cases and dynamic simulation^34–39^. The existing studies have described the construction of regional innovation ecosystems, and revealed the composition of relevant innovation elements. However, when it comes to knowledge diffusion, an especially critical factor in regional innovation ecosystems under digitization, there is still a lack of targeted theoretical research and mathematical validation.

This work considers the issue of promoting knowledge diffusion in regional innovation ecosystems under digitization, and uses the research approach of theoretical model–SEIR model construction–model analysis–model validation–optimal control–strategy exploration, and draws lessons from the epidemic dynamics method in describing the mechanism of knowledge diffusion in systems. By examining the action of digital empowerment, it constructs a susceptible-exposed-infected-recovered (SEIR) model for knowledge diffusion in regional innovation ecosystems under digitization to answer the following questions: (1) How do innovation agents in a regional innovation ecosystem engage in effective knowledge diffusion and dissemination behavior under the drive of digitization? (2) What is the mechanism that drives the innovation agents in a regional innovation ecosystem under digitization to engage in knowledge diffusion?

The research significance of this work is summarized below.Theoretically, it extends the theoretical boundaries of innovation ecosystems and knowledge diffusion. Practically, it offers the management implications for enterprises and governments in decision-making.

## The model

### Theoretical model

**Figure 1:**
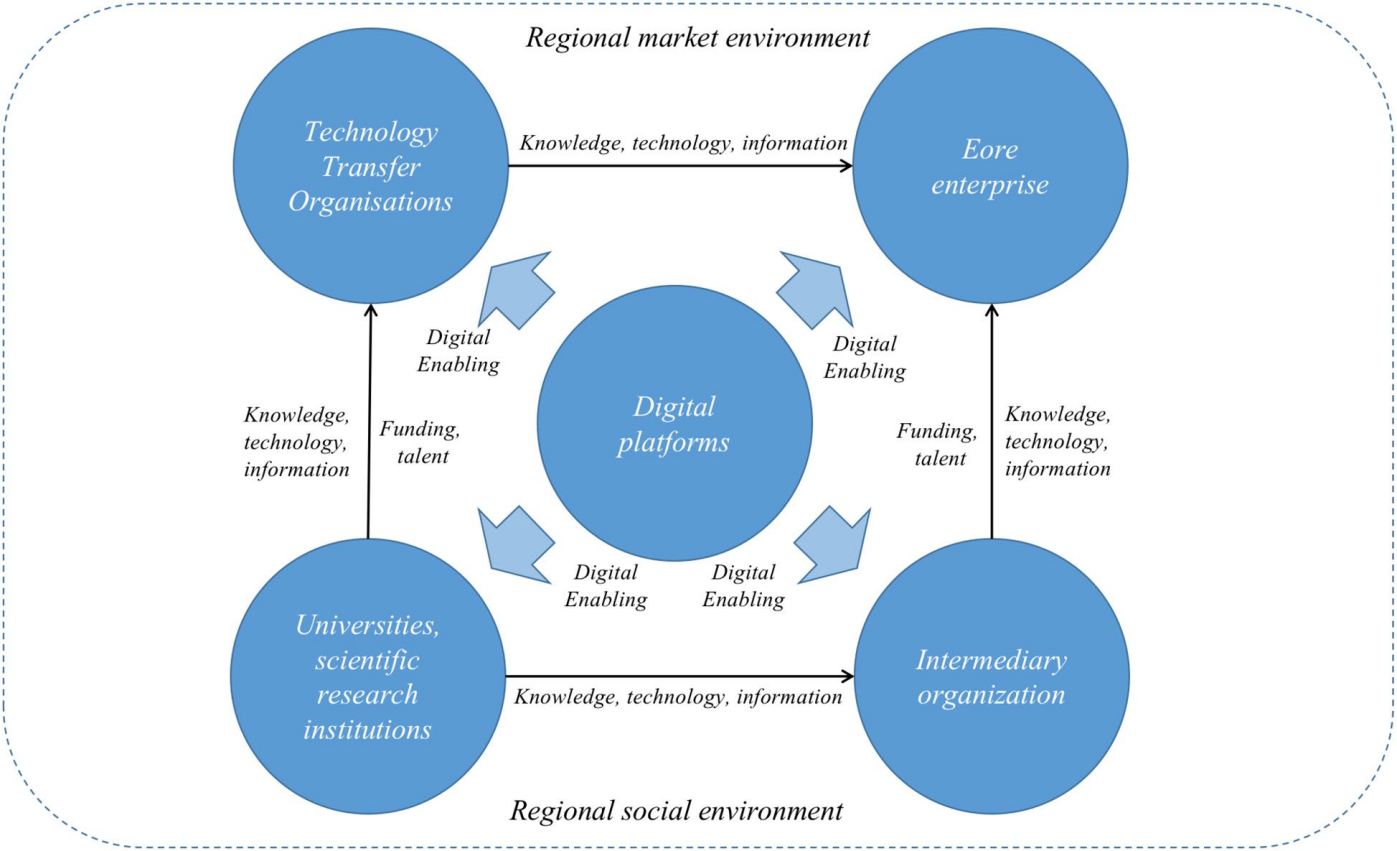
The relationship between the elements of knowledge diffusion in regional innovation ecosystem under digitalization.

In the digital context, the regional innovation ecosystem is no longer confined to time and space constraints^40^ . Instead, relying on digital technologies, critical complementary resources, knowledge-sharing platforms, and knowledge-driven innovation, they restructure the existing innovation resources and processes to achieve the connections and interactions between innovation agents. From the perspective of ecosystems, a regional innovation ecosystem is an open system composed of innovation agents (such as government, core enterprises, universities, research institutions, and intermediaries)^41,42^ and innovation resources (such as knowledge, technology, and information)^43^ . In a regional innovation ecosystem under the combined effect of innovation resources and innovation environments (i.e., market environment and social environment), the innovation agents promote diffusion, dissemination, and innovation of knowledge within the region^44,45^ thereby boosting the evolution and efficiency of knowledge diffusion in the system. Figure 1 illustrates the correlation between the constituent elements of knowledge diffusion in a regional innovation ecosystem under digitization^46^ .

The infectious disease model is one of the micro diffusion models, and research on technology and knowledge diffusion based on infectious disease models has produced many valuable results both domestically and internationally^26^ . At the same time, there are many similarities between the spread of knowledge and the spread and spread of infectious diseases:

1. Possessing ’pathogens’. Pathogens “refer to microorganisms that cause diseases in humans or animals”. The knowledge diffusion in the regional innovation ecosystem drives the dynamic evolution of the system and drives the high-quality development of the regional economy. According to the knowledge complexity attribute of key core technologies, innovative entities need to accumulate rich knowledge in the process of developing and breaking through key core technologies. The disruptive, breakthrough, and timeliness characteristics of key core technologies^47^ promote the diffusion of technology and knowledge within the system in a short period of time, and enable them to be shared and applied. If the system does not attach importance to the iteration and sharing of innovative technology and knowledge, then the innovation entities within the system will be unable to adapt to market demand and will be eliminated by the regional innovation ecosystem. At the same time, the system is influenced by regional innovation capabilities and efficiency, and key core technologies and innovative knowledge will be replaced accordingly. Therefore, the same technology and knowledge will not always be in the core position. With the development of technological innovation, new technologies and knowledge will inevitably be replaced by other new technologies and knowledge at a certain stage. Therefore, in order to enhance regional innovation capacity and efficiency, it is necessary to pay attention to the optimization and upgrading of technology and knowledge in the regional innovation ecosystem, maintain the diffusion process and efficiency of innovative knowledge within the system, and better drive system technology innovation activities.
2. Having “infectivity”. Infectious “refers to the pathogen being expelled from the body through the host and reaching susceptible individuals through certain means” . The susceptible subject in the system completes the infectious effect of “pathogens” by receiving new knowledge. This impact will not end with the susceptible subject, but will continue to spread through the infected subject, achieving the impact of more innovative subjects receiving new knowledge within the system. The contagiousness of regional innovation ecosystems mainly depends on two factors. Firstly, the characteristics of innovation sources themselves, such as the value proposition, value creation, and innovation efficiency of innovation entities. Secondly, the receptive ability of susceptible individuals to new knowledge. This includes subjective acceptance of new technological knowledge and a certain resource base.
3. Having “immunity”. Immunity “refers to the high resistance of the body to disease infection. In the infectious disease model, compared to those who have already been cured, they have strong immunity to pathogens”. In the knowledge diffusion model of regional innovation ecosystem under digitization, the process of selecting a new knowledge and accepting it by the innovation subject is considered as pathogen infection. However, when the innovation subject gives up infecting a new knowledge and chooses another alternative knowledge, it can be said that the innovation subject is cured. The alternative knowledge is more progressiveness than the original knowledge, so the innovative subject who chooses the alternative knowledge will not choose the original knowledge again to learn. At this point, the innovative subject has strong immunity and will not be re infected by the same pathogen.

Transfer key concepts from the infectious disease model to the knowledge diffusion model, as shown in Table 1:

**Table 1:** The conceptual transfer of knowledge diffusion models in Regional Innovation Ecosystems under the model of infectious diseases and digitalization.

SIR model	SEIR model	Knowledge diffusion model
Pathogen	The knowledge diffused in the regional innovation ecosystem under digitization	Innovation knowledge diffusion
Carriers	Subjects holding innovative knowledge	Innovation source
Susceptible person	Potential diffusion knowledge subjects with diffusion ability in the system	Potential knowledge diffusion subjects
Infected people	Subjects involved in knowledge diffusion	Recipient
Removed people	Received the knowledge but used alternative knowledge	Immune person
Infection rate	Ratio of knowledge diffusion entities receiving innovative knowledge	Diffusivity
Cure rate	Ratio of innovative entities using alternative knowledge	Abandonment rate

### SEIR model

In the regional innovation ecosystem, the knowledge of each innovation subject mainly comes from two aspects: Firstly, the innovation subject relies on their own knowledge, technology, patents, etc., which mainly comes from the subject’s own experience accumulation;Secondly, the interaction mechanism between innovative entities in the system leads to the diffusion and dissemination of new knowledge, which mainly originates from the linkage and transmission of innovative elements between entities.Due to the diffusion of knowledge, there is a dependency relationship between innovation entities to acquire knowledge.Therefore, this article establishes a knowledge diffusion model for regional innovation ecosystems under digitization, where the scale of entities is variable at any point at time *t*, and the total scale of entities can be represented by *N*(*t*). This article does not currently consider the complexity of human subjective consciousness and the heterogeneity of human behavioral factors in the system. In the regional innovation ecosystem, innovation subject can be divided into four categories: (1) *S*(*t*) represents the knowledge holder. Innovation subject in the regional innovation ecosystem who possess a certain type of innovation knowledge. (2) *E*(*t*) represents the potential knowledge diffusion subject. In the regional innovation ecosystem, innovation subject have mastered a certain type of innovation knowledge, but currently do not have the willingness or ability to diffuse that knowledge. (3) *I*(*t*) represents the subject of knowledge diffusion. In the regional innovation ecosystem, innovation subject have mastered a certain type of innovation knowledge and possess the willingness and ability to spread knowledge. (4) *R*(*t*) represents a knowledge immune agent. In the regional innovation ecosystem, the innovation subject uses alternative knowledge, so knowledge diffusion is not carried out.

In the constructed model, digital platforms drive the interaction, diffusion, and dissemination of knowledge, technology, information, and other innovative resources among the innovation subject of the regional innovation ecosystem, thereby enhancing the innovation capacity and efficiency of the ecosystem. However, in the system, some subjects do not currently have the support of digital tools and channels, resulting in differences in their understanding or learning level of new knowledge. At this time, the knowledge diffusion subject is in a weak state of knowledge-diffusion ability. Therefore, some subjects are unable to effectively spread knowledge.

**Figure 2:**
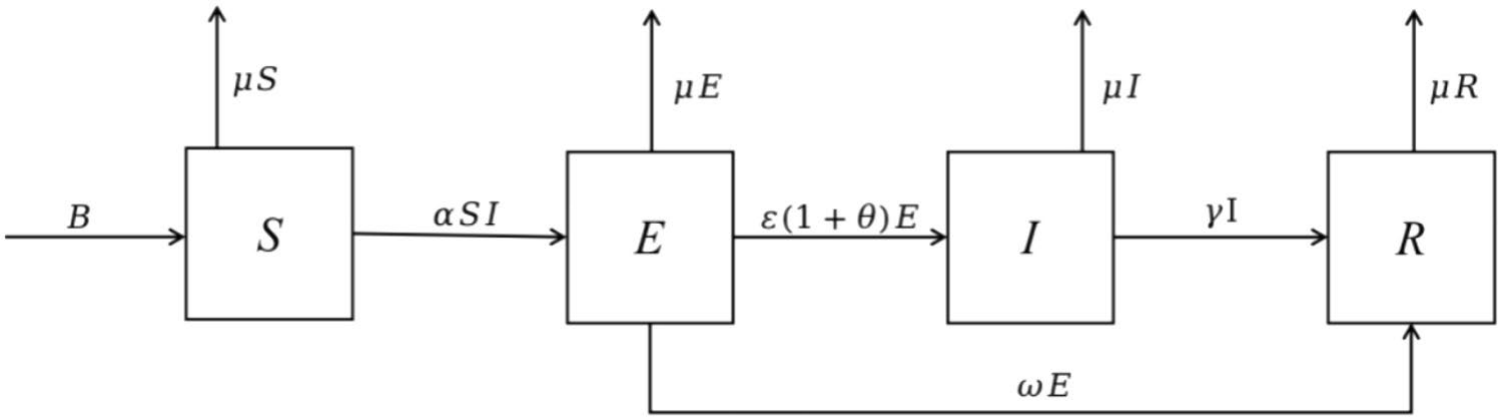
The flow diagram of the model.

Based on the above analysis, this article uses the SEIR model in the infectious disease model to explore the knowledge diffusion mechanism of regional innovation ecosystems under digitization. The model flowchart is shown in Fig. 2.

The parameters in the SEIR model are explained as follows:

1. The number of innovation entities in a regional innovation ecosystem typically changes over time. Therefore, this article defines *B* as the number of innovation agents entering the regional innovation ecosystem. At the same time, over time, the innovative subjects in the system may emerge and disappear due to various force majeure factors and the decline of the ecosystem lifecycle, with the removal rate recorded as $\mu$.
2. When knowledge diffusion occurs within a regional innovation ecosystem, the knowledge receiving entity will have a certain contact rate with potential knowledge diffusion subjects, and the contact rate will be recorded as.$\alpha$
3. For subjects with the willingness and ability to spread knowledge, a portion of the knowledge receiving subjects will rely on digital platforms, digital technologies, industrial alliances, etc. to drive the depth and breadth of new knowledge diffusion, denoted as $\theta$. At the same time, this type of knowledge diffusion agent will accelerate the efficiency of knowledge diffusion, and the conversion rate of the knowledge diffusion agent is recorded as $(1+\theta )$.
4. In the evolution process of regional innovation ecosystem under digitization, some potential knowledge diffusion entities transform from potential knowledge diffusion entities to knowledge diffusion entities with $\varepsilon$ probability after building digital platforms, accumulating their own practical experience, and absorbing knowledge through patent applications; At the same time, if the subject receives the knowledge but uses alternative knowledge, it will be transformed into a knowledge immune subject with a $\omega$ probability.
5. When the knowledge diffusion subject uses substitute knowledge after a series of learning, internalization, and absorption processes on a certain innovative knowledge, the original knowledge will no longer need to be diffused and will be transformed into a knowledge immune subject with a $\gamma$ probability.

The parameters of *SEIR* model are summarized in Table 2.

**Table 2:** The parameter description of SEIR Model.

Parameter	Description
*S*(*t*)	The number of innovative knowledge holders at the time *t*.
*E*(*t*)	The number of potential knowledge diffusion subjects at the time *t*.
*I*(*t*)	The number of innovative subjects that diffuse existing knowledge at the time *t*.
*R*(*t*)	The number of knowledge immunization innovation subject at the time *t*.
$\alpha$	Knowledge diffusion rate.
$\theta$	The ratio of potential knowledge diffusion agents with digital transmission capability to the number of
	knowledge diffusion agents.
$\varepsilon$	Transition probability from state *E* to state *I*.
$\omega$	Transition probability from state *E* to state *R*.
${\gamma }$	Transition probability from state *I* to state *R*.
$\mu$	Removal rate per unit time.
*B*	The number of immigrants in the regional innovation ecosystem per unit time.

Based on the above analysis, we construct an *SEIR* model for knowledge diffusion in regional innovation ecosystem under digitization. The system dynamics equations are described as follows:

1 $$\begin{aligned} \begin{aligned} \ \left\{ \begin{array}{l} \frac{{dS}}{{dt}} = B - \alpha SI - \mu S,\\ \frac{{d{E}}}{{dt}} = \alpha SI - \varepsilon (1 + \theta ) {E} - {\omega }{E} - \mu {E},\\ \frac{{dI}}{{dt}} = \varepsilon (1 + \theta ) {E} - \gamma I - \mu I,\\ \frac{{dR}}{{dt}} = \gamma I + {\omega }{E} - \mu R. \end{array} \right. \ \end{aligned} \end{aligned}$$

where:

2 $$\begin{aligned} \begin{aligned} \ B> 0,\mu> 0,\alpha> 0,\varepsilon> 0,\omega> 0,\gamma > 0,\theta \in (0,1],\ \end{aligned} \end{aligned}$$

and

3 $$\begin{aligned} \begin{aligned} \ S(t) + {E}(t) + I(t) + R(t) = N(t).\ \end{aligned} \end{aligned}$$

## Stability analysis of the model

Firstly, the basic reproduction number $R_0$ of system (1) approach in the next generation matrix^48^. In this paper, $R_0$ represents the number of next generation through knowledge diffusion.

Let $X=(E,I,R,S)^T$, then system (1) can be written as:

4 $$\begin{aligned}{} & {} \begin{aligned} \frac{{dX}}{{dt}} = F(X) - V(X), \end{aligned} \end{aligned}$$

5 $$\begin{aligned}{} & {} \begin{aligned} \ F(X) = \left( {\begin{array}{*{20}{c}} {\alpha SI}\\ {\varepsilon (1 + \theta ) {E}}\\ 0\\ \begin{array}{l} 0\\ \end{array} \end{array}} \right) ,V(X) = \left( {\begin{array}{*{20}{l}} {\varepsilon (1 + \theta ) {E} + {\omega }{E} + \mu {E}}\\ {\gamma I + \mu I}\\ \begin{array}{l} - \gamma I - \omega {E} + \mu {R}\\ - B + \alpha SI + \mu S \end{array} \end{array}} \right) .\ \end{aligned} \end{aligned}$$

Calculate the Jacobian matrices of *F*(*X*) and *V*(*X*) in system (5) respectively, and then take the sub matrices corresponding to the first two variables (i.e. E, I) directly related to the number of knowledge diffusion. The results are as follows:

6 $$\begin{aligned} \begin{aligned} \ F = \left( {\begin{array}{*{20}{l}} 0&{}\quad {\alpha S}\\ \varepsilon (1+\theta ) &{}\quad 0 \end{array}} \right) ,V = \left( {\begin{array}{*{20}{l}} {\varepsilon (1+\theta ) + {\omega } + \mu }&{}\quad 0\\ 0&{}\quad {\gamma \ + \mu } \end{array}} \right) ,\ \end{aligned} \end{aligned}$$

where *F* and *V* represent the infection and transition matrices respectively^38^.By simple calculation, the inverse matrix of V can be obtained as:

7 $$\begin{aligned} \begin{aligned} \ {V^{-1}} = \left( {\begin{array}{*{20}{l}} {\frac{1}{\varepsilon (1+\theta ) + \omega + \mu }}&{}\quad 0\\ 0 &{}\quad {\frac{1}{\gamma + \mu }} \end{array}} \right) .\ \end{aligned} \end{aligned}$$

The next generation matrix is:

8 $$\begin{aligned} \begin{aligned} \ {FV^{-1}} = \left( {\begin{array}{*{20}{l}} 0&{}\quad {\frac{\alpha S}{\gamma + \mu }}\\ {\frac{\varepsilon (1+\theta )}{\varepsilon (1+\theta ) + \omega + \mu }}&{}\quad 0 \end{array}} \right) .\ \end{aligned} \end{aligned}$$

Hence, the basic reproduction number $R_0$ of system (1) is the spectral radius of the next generation matrix $F{V^ {-1} }$. Here, the spectral radius is the maximum value of characteristic root of $F{V^ {-1} }$. Therefore, $R_0$ can be computed as:

9 $$\begin{aligned} \begin{aligned} \ {R_0} = \rho (F{V^ {-1} }) = \sqrt{\frac{{B\alpha \varepsilon (1 + \theta )}}{{\mu [\varepsilon (1 + \theta ) + \omega + \mu ](\gamma + \mu )}}}.\ \end{aligned} \end{aligned}$$

Then, the knowledge will disappear if $R_0<1$, and the knowledge-free equilibrium point of system (1) can be easily observed as ${E^0} = \left( B / \mu ,0,0,0 \right)$.

While the knowledge will be diffuse if $R_0>1$, and the knowledge-existence equilibrium point of system (1) can be obtained as ${E^*} = \left( {{S^*},E^*,{I^*},{R^*}} \right)$. ${E^*}$ must satisfy the following equations :

10 $$\begin{aligned} \begin{aligned} \ \left\{ \begin{array}{l} B - \alpha {S^*}{I^*} - \mu {S^*} = 0,\\ \alpha {S^*}{I^*} - \varepsilon (1+\theta ) {{E^*}} - {\omega }{{E^*}} - \mu {{E^*}} = 0,\\ \varepsilon (1+\theta ) {{E^*}} - \gamma {I^*} - \mu {I^*} = 0,\\ \gamma {I^*} + {\omega }{{E^*}} - \mu {R^*} = 0. \end{array} \right. \ \end{aligned} \end{aligned}$$

Solving the equations of Eq. (10), we can get the knowledge-existence equilibrium point ${E^*} = \left( {{S^*},{E^*},{I^*},{R^*}} \right)$ :

11 $$\begin{aligned}{} & {} \begin{aligned} \ {S^*} = \frac{B}{{\alpha I^* + \mu }},\ \end{aligned} \end{aligned}$$

12 $$\begin{aligned}{} & {} \begin{aligned} \ E^* = \frac{{ \alpha (1+\theta ) B - \mu (\gamma + \mu )[(1+\theta )+\omega +\mu ] }}{{\alpha (1 + \theta )[\varepsilon (1+\theta )+\omega +\mu ]}},\ \end{aligned} \end{aligned}$$

13 $$\begin{aligned}{} & {} \begin{aligned} \ I^* = \frac{{\alpha \varepsilon (1+\theta ) B - \mu (\gamma +\mu )[\varepsilon (1+\theta )+\omega +\mu ]}}{{\alpha (\gamma +\mu )[\varepsilon (1+\theta )+\omega +\mu ])}},\ \end{aligned} \end{aligned}$$

14 $$\begin{aligned}{} & {} \begin{aligned} \ {R^*} = \frac{{\gamma I^* + \omega E^*}}{{\mu }}.\ \end{aligned} \end{aligned}$$

### Theorem 1

If $R_0<1$ and $[\varepsilon (1+\theta )+\omega +\mu ](\gamma +\mu ) > \frac{\varepsilon (1+\theta )\alpha B}{\mu }$,the knowledge-free equilibrium point ${E^0} \!=\! \left( B \mu ,0,0,0 \right)$ of system (1) is locally asymptotically stable.

### Proof of Theorem 1

The Jacobin matrix of system (1) at knowledge-free equilibrium point ${E^0} = \left( B \mu ,0,0,0 \right)$ can be written as:

15 $$\begin{aligned} \begin{aligned} \ J({E^0}) = \left[ {\begin{array}{*{20}{c}} { - \mu }&{}0&{}{ - \frac{\alpha B}{\mu }}&{}0\\ 0&{}{ -\varepsilon (1+\theta )-\omega -\mu }&{}{\frac{\alpha B}{\mu }}&{}0\\ 0&{}\varepsilon (1+\theta ) &{}{ - {\gamma } - \mu }&{}0\\ 0&{}\omega &{}{ \gamma }&{}{-\mu }\\ \end{array}} \right] .\ \end{aligned} \end{aligned}$$

It is easy to know negative eigenvalues of $J({E^0})$ ${\Lambda _{01}} = {\Lambda _{02}} = - \mu < 0$, and the other eigenvalues are the characteristic roots of $\left| {hE - J({E^0})} \right|$, where:

16 $$\begin{aligned} \begin{aligned} \ \left| {hE - J({E^0})} \right| = \left| {\begin{array}{*{20}{c}} {h + \varepsilon (1+\theta )+\omega +\mu }&{}{ - \frac{\alpha B}{\mu }}\\ { - \varepsilon (1+\theta ) }&{}{h + \gamma + \mu } \end{array}} \right| =0.\ \end{aligned} \end{aligned}$$

The eigenvalues of Eq. (16) can be obviously obtained as:

17 $$\begin{aligned} \begin{aligned} \ \begin{array}{*{20}{l}} {{\Lambda _{03}}}= & {} {\frac{{-[\gamma +2\mu +\varepsilon (1+\theta )+\omega ] -\sqrt{{{[\gamma + 2\mu + \varepsilon (1+\theta ) + \omega ]}^2}} - 4[(\varepsilon (1+\theta )+\omega +\mu )(\gamma +\mu )-\frac{\varepsilon (1+\theta \alpha B)}{\mu }]} }{2},} \end{array}\ \end{aligned} \end{aligned}$$

and

18 $$\begin{aligned} \begin{aligned} \ \begin{array}{*{20}{l}} {{\Lambda _{04}}}= & {} {\frac{{-[\gamma +2\mu +\varepsilon (1+\theta )+\omega ] -\sqrt{{{[\gamma + 2\mu + \varepsilon (1+\theta ) + \omega ]}^2}} + 4[(\varepsilon (1+\theta )+\omega +\mu )(\gamma +\mu )-\frac{\varepsilon (1+\theta \alpha B)}{\mu }]} }{2},} \end{array}\ \end{aligned} \end{aligned}$$

Because all parameters of system (1) are non negative.Obviously, ${\Lambda _{01}} < 0$ and ${\Lambda _{02}} < 0$ .when $[\varepsilon (1+\theta )+\omega +\mu ](\gamma +\mu ) > {\frac{\varepsilon (1+\theta )\alpha B}{\mu }}$, so ${\Lambda _{03}} < 0$. Hence, the knowledge-free equilibrium point ${E^0} = \left( B\mu ,0,0,0 \right)$ of system (1) is locally asymptotically stable if $R_0<1$ based on the Routh-Hurwitz criterion.

### Theorem 2

If $R_0>1$, and $[\varepsilon (1+\theta )+\omega +\mu ](\gamma +\mu ) > {\frac{\varepsilon (1+\theta )\alpha B}{\mu }}$, the knowledge-existence equilibrium point ${E^*} = ( {S^*},{E^*},{I^*},{R^*})$ of system (1) is locally asymptotically stable.

### Proof of Theorem 2

The Jacobin matrix of system (1) at knowledge-existence equilibrium point ${E^*} = \left( {{S^*},{E^*},{I^*},{R^*}} \right)$ can be written as:

19 $$\begin{aligned} \begin{aligned} \ J({E^*}) =\left[ {\begin{array}{*{20}{l}} {- \alpha {I^*}}&{}\quad \!0&{}\quad {-\alpha {S^*}}&{}\quad \!0\\ {\alpha {I^*}}&{}\quad -\varepsilon (1+\theta )-\omega -\mu &{}\quad {\alpha {S^*}}&{}\quad 0\\ {0}&{}\quad { \varepsilon (1+\theta )}&{}\quad {- {\gamma }-\mu }&{}\quad 0\\ 0&{}\quad \omega &{}\quad {\gamma }&{}\quad {-\mu }\\ \end{array}} \right] .\ \end{aligned} \end{aligned}$$

It is easy to know that two of the negative eigenvalues of system (19) are ${\Lambda _{11}} = - \mu$, and the other eigenvalues are the characteristic roots of $\left| {hE - J({E^*})} \right|$, where:

20 $$\begin{aligned} \begin{aligned} \ \left| {hE \!-\! J({E^*})} \right| \!=\! \left| {\begin{array}{*{20}{l}} {h+\alpha {I^*} }&{}\quad 0&{}\quad {\alpha {S^*}}\\ {-\alpha {I^*}}&{}\quad {h+\varepsilon (1+\theta )+\omega +\mu }&{}\quad {-\alpha {S^*}}\\ 0&{}{-\varepsilon (1+\theta ) }&{}{h+\gamma +\mu } \end{array}} \right| .\ \end{aligned} \end{aligned}$$

We can obviously obtain the eigenvalues of Eq. (20), where:

21 $$\begin{aligned} \begin{aligned} \ \begin{array}{*{20}{l}} {\left| {hE - J({E^*})} \right| }&{}\mathrm{{ = }}&{}{{h^3} + [(\gamma +2\mu +\varepsilon (1+\theta )+\omega +\alpha I^* ]{h^2}}\\ {}&{}\quad \mathrm{{ + }}&{}{[\varepsilon (1+\theta )\gamma +\varepsilon (1+\theta )\mu +\omega \gamma +\omega \mu +\gamma \mu +\mu ^2}\\ {}&{}\quad \mathrm{{ + }}&{}{\alpha I^*\gamma +\alpha I^*\mu +\alpha I^*\varepsilon (1+\theta )+\alpha I^*\omega +\alpha I^*-\alpha S^*\varepsilon (1+\theta )]h }\\ {}&{}\quad \mathrm{{ + }}&{}{[\alpha I^*\varepsilon (1+\theta )\gamma + \alpha I^* \varepsilon (1+\theta )\mu +\alpha I^*\omega \gamma +\alpha I^*\omega \mu }\\ {}&{}\quad \mathrm{{ + }}&{}{\alpha I^*\mu \gamma +\alpha I^*\mu ^2].} \end{array}\ \end{aligned} \end{aligned}$$

Then we construct a cubic polynomial and replace the coefficient with ${a_3},{a_2},{a_1},{a_0}$ to determine the other eigenvalues of system (19). Hence, Eq. (21) can be rewritten as:

22 $$\begin{aligned} \begin{aligned} \ {a_3}{h^3} + {a_2}{h^2} + {a_1}h + {a_0} = 0,\ \end{aligned} \end{aligned}$$

where:

23 $$\begin{aligned}{} & {} \begin{aligned} \ {a_3} = 1,\ \end{aligned} \end{aligned}$$

24 $$\begin{aligned}{} & {} \begin{aligned} \ {a_2} = \gamma + 2\mu +\varepsilon (1+\theta )+\omega +\alpha I^*,\ \end{aligned} \end{aligned}$$

25 $$\begin{aligned}{} & {} \begin{aligned}{}&\ {a_1} = \varepsilon (1+\theta )\gamma +\varepsilon (1+\theta )\mu +\omega \gamma +\omega \mu +\gamma \mu +\mu ^2+\alpha I^*\gamma +\alpha I^*\mu \\&\quad \quad +\alpha I^*\varepsilon (1+\theta )+\alpha I^*\omega +\alpha I^*-\alpha S^* \varepsilon (1+\theta ),\ \end{aligned} \end{aligned}$$

26 $$\begin{aligned}{} & {} \begin{aligned}{}&\ {a_0} = \alpha I^*\varepsilon (1+\theta )\gamma \mathrm{{ + }}{\alpha I^*\varepsilon (1+\theta )\mu }\mathrm{{ + }}\alpha I^*\omega \gamma \mathrm{{ + }}\alpha I^*\omega \mu \mathrm{{ + }}\alpha I^*\mu \gamma \mathrm{{ + }}\alpha I^*\mu ^2,\ \end{aligned} \end{aligned}$$

and

27 $$\begin{aligned} \begin{aligned} \ \begin{array}{*{20}{l}} {{a_2}{a_1} - {a_3}{a_0}}&{} = &{}{[\gamma + 2\mu +\varepsilon (1+\theta )+\omega +\alpha I^*][\varepsilon (1+\theta )\gamma +\varepsilon (1+\theta )\mu +\omega \gamma +\omega \mu +\gamma \mu }\\ {}&{}\quad + &{}\mu ^2+\alpha I^*\gamma +\alpha I^*\mu +\alpha I^*\varepsilon (1+\theta )+\alpha I^*\omega +\alpha I^*-\alpha S^*\varepsilon (1+\theta )]\\ {}&{}\quad - &{}[\alpha I^*\varepsilon (1+\theta )\gamma +\alpha I^*\varepsilon (1+\theta )\mu +\alpha I^*\omega \gamma +\alpha I^* \omega \mu +\alpha I^*\mu \gamma +\alpha I^*\mu ^2]. \end{array}\ \end{aligned} \end{aligned}$$

The condition of knowledge-existence equilibrium point ${E^*} = \left( {{S^*},E^*,{I^*},{R^*}} \right)$ is locally asymptotically stable and the conditions:(i) ${a_3},{a_2},{a_1},{a_0} > 0$ and (ii) ${a_2}{a_1} - {a_3}{a_0} > 0$ based on the Routh-Hurwitz criterion. It is easy to know that ${a_3},{a_2},{a_0} > 0$.

If ${\alpha }B > \varepsilon \gamma \mu$ and ${R_0} > 1$, then ${a_1} > 0$ and ${a_2}{a_1} - {a_3}{a_0} > 0$. In this case, the Routh-Hurwitz criterion are satisfied. Hence, the knowledge-existence equilibrium point ${E^*} = \left( {{S^*},E^*,{I^*},{R^*}} \right)$ of system (1) is locally asymptotically stable.

## The optimal control model

Based on the above knowledge diffusion model, we propose two control objectives to expand the scope of knowledge diffusion. On the one hand, the more subjects of knowledge diffusion, the larger the scope of knowledge diffusion.On the other hand, knowledge diffusion entities have more digital channels, which can better achieve knowledge diffusion within the regional innovation ecosystem.

Hence, the two proportion constants $\varepsilon$ and $\theta$ in the model are transformed into control variables $\varepsilon (t)$ and $\theta (t)$. The control variable $\varepsilon (t)$ is used to control the proportion of authoritative “Knowledge diffusion subject” in the crowd. The potential diffusion subjects in the regional innovation ecosystem rely on the support of innovation environments such as industry university research innovation alliances, intermediary agencies, and government policies to guide the transformation of potential knowledge diffusion entities into knowledge diffusion subjects. Control variable $\theta (t)$ is used to control the proportion of knowledge holders with digital transmission capabilities.The regional innovation ecosystem enhances the scope and efficiency of knowledge diffusion through digital construction of office systems such as OA, ERP, and industrial internet platforms. At the same time, the innovation entities of the regional innovation ecosystem will rely on digital technology to enhance their knowledge diffusion capabilities. In this model, the control variables $0\le \varepsilon \left( t\right) \le 1$ and $0\le \theta \left( t\right) \le 1$. While $\varepsilon \left( t\right) =1$ and $\theta \left( t\right) =1$, it means that the control effect is optimal and the knowledge can be diffused to the greatest extent. On the contrary, while $\varepsilon \left( t\right) =0$ and $\theta \left( t\right) =0$, it means that the control measures are completely ineffective.

Hence, we propose an objective function as:

28 $$\begin{aligned} \begin{aligned} \ J(\varepsilon ,\theta ) = \int \limits _0^{{t_f}} {\left[ {{E}(t) + I(t) - \frac{{{c_1}}}{2}{\varepsilon ^2}(t) - \frac{{{c_2}}}{2}{\theta ^2}(t)} \right] } dt,\ \end{aligned} \end{aligned}$$

and satisfy the state system as:

29 $$\begin{aligned} \begin{aligned} \ \left\{ \begin{array}{l} \frac{{dS}}{{dt}} = B - \alpha SI - \mu S,\\ \frac{{d{E}}}{{dt}} = \alpha SI + \varepsilon (t)(1+\theta (t)) {E} - {\omega }{E} - \mu {E},\\ \frac{{dI}}{{dt}} = \varepsilon (t)(1+\theta (t)) {E} - \gamma I - \mu I,\\ \frac{{dR}}{{dt}} = \gamma I + {\omega }{E} - \mu R. \end{array} \right. \ \end{aligned} \end{aligned}$$

The initial conditions for system (29) are satisfied:

30 $$\begin{aligned} \begin{aligned} \ S(0) = {S_0},{E}(0) = {E_{0}},I(0) = {I_0},R(0) = {R_0},\ \end{aligned} \end{aligned}$$

where:

31 $$\begin{aligned} \begin{aligned} \ \varepsilon (t),\theta (t) \in U \buildrel \Delta \over = \left\{ {\left. {(\varepsilon ,\theta )} \right| (\varepsilon (t),\theta (t))measurable,0 \le \varepsilon (t),\theta (t) \le 1,\forall t \in \left[ {0,{t_f}} \right] } \right\} ,\ \end{aligned} \end{aligned}$$

while *U* is the admissible control set. The time interval of control is between 0 and $t_f$. $c_1$ and $c_2$ are positive weight coefficients shown the control strength and importance of two control measures.

### Theorem 3

An optimal control pair $({\varepsilon ^*},{\theta ^*}) \in U$ exists so that the function is established below:

32 $$\begin{aligned} \begin{aligned} \ J({\varepsilon ^*},{\theta ^*}) = \max \left\{ {J(\varepsilon ,\theta ):(\varepsilon ,\theta ) \in U} \right\} .\ \end{aligned} \end{aligned}$$

### Proof of Theorem 3

Let $$X(t) = {(S(t),{E}(t),I(t),R(t))^T}$$ and

33 $$\begin{aligned} \begin{aligned} \ L(t;X(t),\varepsilon (t),\theta (t)) = {E}(t) + I(t) - \frac{{{c_1}}}{2}{\varepsilon ^2}(t) - \frac{{{c_2}}}{2}{\theta ^2}(t).\ \end{aligned} \end{aligned}$$

The existence of an optimal pair must satisfy: (i) the set of control variables and state variables is nonempty, (ii) the control set *U* is convex and closed, (iii) the right-hand side of the state system is bounded by a linear function in the state and control variables, (iv) the integrand of the objective functional is convex on *U*, (v) there exist constants ${d_1},{d_2} > 0$ and $\rho > 1$ such that the integrand of the objective functional satisfies:

34 $$\begin{aligned} \begin{aligned} \ - L(t;X(t),\varepsilon ;\theta ) \ge {d_1}{\left( {{{\left| \varepsilon \right| }^2} + {{\left| \theta \right| }^2}} \right) ^{{\rho /2}}} - {d_2}.\ \end{aligned} \end{aligned}$$

Conditions (i)–(iii) is clearly established, we just prove the condition (iv) and (v). One can easily obtain inequality:

35 $$\begin{aligned} \begin{aligned} \ S' \le B,{E}^\prime \!\le \! \alpha SI,I' \!\le \! \varepsilon (t)(1+\theta (t)){E},R' \!\le \! \gamma I \!+\! {\omega }{E} \!.\ \end{aligned} \end{aligned}$$

Hence, condition (iv) is established. Then, for any $t \ge 0$, there is a positive constant *M* which is satisfied $\left| {X(t)} \right| \le M$, therefore

36 $$\begin{aligned} \begin{aligned} \ - L(t;X(t),\varepsilon ;\theta ) = \frac{{{c_1}{\varepsilon ^2}(t) + {c_2}{\theta ^2}(t)}}{2} - {E}(t) - I(t) \ge {d_1}{\left( {{{\left| \varepsilon \right| }^2} + {{\left| \theta \right| }^2}} \right) ^{{\rho /2}}} - 2M.\ \end{aligned} \end{aligned}$$

Let ${d_1} = \min \left\{ {\frac{{{c_1}}}{2},\frac{{{c_2}}}{2}} \right\} ,{d_2} = 2M$ and $\rho = 2$, then condition (v) is established. Hence, the optimal control can be realized.

### Theorem 4

For the optimal control pair $({\varepsilon ^*},{\theta ^*})$ of state system (29), there exist adjoint variables ${\delta _1},{\delta _2},{\delta _3},{\delta _4}$ that satisfy:

37 $$\begin{aligned} \begin{aligned} \ \left\{ \begin{array}{l} \frac{{d{\delta _1}}}{{dt}} = ({\delta _1} - {\delta _2})\alpha I + {\delta _1}\mu ,\\ \frac{{d{\delta _2}}}{{dt}} = 1 + ({\delta _2} - {\delta _3})\varepsilon (t)(1+\theta (t)) + ({\delta _2} - {\delta _4}){\omega } - {\delta _2}\mu ,\\ \frac{{d{\delta _3}}}{{dt}} = 1 + ({\delta _1} - {\delta _2})\alpha S + {\delta _3} (\gamma +\mu ) - {\delta _4}\gamma ,\\ \frac{{d{\delta _4}}}{{dt}} = {\delta _4}\mu . \end{array} \right. \ \end{aligned} \end{aligned}$$

With boundary conditions:

38 $$\begin{aligned} \begin{aligned} \ {\delta _1}({t_f}) = {\delta _2}({t_f}) = {\delta _3}({t_f}) = {\delta _4}({t_f}) = 0.\ \end{aligned} \end{aligned}$$

In addition, the optimal control pair $({\varepsilon ^*},{\theta ^*})$ of state system (29) can be given by:

39 $$\begin{aligned}{} & {} \begin{aligned} \ {\varepsilon ^*}(t) = \min \left\{ {1,\max \left\{ {0,\frac{{({\delta _2} - {\delta _3}) (1+\theta (t))E}}{{{c_1}}}} \right\} } \right\} ,\ \end{aligned} \end{aligned}$$

40 $$\begin{aligned}{} & {} \begin{aligned} \ {\theta ^*}(t) = \min \left\{ {1,\max \left\{ {0,\frac{{({\delta _2} - {\delta _3}) \varepsilon (t)}}{{{c_2}}}} \right\} } \right\} .\ \end{aligned} \end{aligned}$$

### Proof of Theorem 4

Define a Hamiltonian function enlarged with penalty term to obtain the expression of optimal control system and optimal control pair. The Hamiltonian function enlarged can be written as:

41 $$\begin{aligned} \begin{aligned} \ \begin{array}{*{20}{l}} H&={ - {E}(t) - I(t) + \frac{{{c_1}}}{2}{\varepsilon ^2}(t) + \frac{{{c_2}}}{2}{\theta ^2}(t) + {\delta _1}[B - \alpha SI - \mu S]}\\ &\quad +{{\delta _2}[\alpha SI \!-\! \varepsilon (t) (1+\theta (t)){E}- {\omega }{E}- \mu {E}] + {\delta _3}[\varepsilon (t)(1+\theta (t))E -\gamma I-\mu I ]}\\ &\quad + {{\delta _4}[\gamma I + \omega E - \mu R] }\\ &\quad -{{\phi _{11}}\varepsilon (t) - {\phi _{12}}(1-\varepsilon (t))-\phi _{21}\theta (t)-\phi _{22}(1-\theta (t)),} \end{array}\ \end{aligned} \end{aligned}$$

which the penalty term is ${\phi _{ij}}(t) \ge 0$ , and it is satisfied that ${\phi _{11}}(t)\varepsilon (t) = {\phi _{12}}(t)(1 - \varepsilon (t)) = 0$ at optimal control ${\varepsilon ^*}$ and ${\phi _{21}}(t)\theta (t) = {\phi _{22}}(t)(1 - \theta (t)) = 0$ at optimal control ${\theta ^*}$.

Based on the Pontyragin maximum principle, the adjoint system can be written as:

42 $$\begin{aligned} \begin{aligned} \ \frac{{d{\delta _1}}}{{dt}} = - \frac{{\partial H}}{{\partial S}},\frac{{d{\delta _2}}}{{dt}} = - \frac{{\partial H}}{{\partial {E}}},\frac{{d{\delta _3}}}{{dt}} = - \frac{{\partial H}}{{\partial {I}}},\frac{{d{\delta _4}}}{{dt}} = - \frac{{\partial H}}{{\partial R}},\ \end{aligned} \end{aligned}$$

and the boundary conditions of adjoint system are

43 $$\begin{aligned} \begin{aligned} \ {\delta _1}({t_f}) = {\delta _2}({t_f}) = {\delta _3}({t_f}) = {\delta _4}({t_f}) = 0.\ \end{aligned} \end{aligned}$$

Let ${\varepsilon ^*}$ as an example to give the optimality conditions. One have

44 $$\begin{aligned} \begin{aligned} \ \frac{{\partial H}}{{\partial \varepsilon }} = {c_1}\varepsilon (t) - {\delta _2}(1+\theta (t))E + {\delta _3}(1+\theta (t))E - {\phi _{11}} + {\phi _{12}} = 0,\ \end{aligned} \end{aligned}$$

and the optimal control formulae can be written as:

45 $$\begin{aligned} \begin{aligned} \ {\varepsilon ^*} = \frac{{({\delta _2} - {\delta _3})(1+\theta (t))E}}{{{c_1}}} + {\phi _{11}} - {\phi _{12}}.\ \end{aligned} \end{aligned}$$

To obtain the final optimal control formulae without ${\phi _{11}}$ and ${\phi _{12}}$ need to consider the following three situations.

The first situation is that ${\phi _{11}}(t) = {\phi _{12}}(t) = 0$ in set $\left\{ {\left. t \right| 0< {\varepsilon ^*}(t) < 1} \right\}$, then the optimal control formulae can be written as:

46 $$\begin{aligned} \begin{aligned} \ {\varepsilon ^*}(t) = \frac{1}{{{c_1}}}({\delta _2} - {\delta _3})(1+\theta (t))E.\ \end{aligned} \end{aligned}$$

The second situation is that ${\phi _{11}}(t) = 0$ in set $\left\{ {\left. t \right| {\varepsilon ^*}(t) = 1} \right\}$, then the optimal control formulae can be written as:

47 $$\begin{aligned} \begin{aligned} \ 1 = {\varepsilon ^*}(t) = \frac{1}{{{c_1}}}[(1+\theta (t))E - {\phi _{12}}].\ \end{aligned} \end{aligned}$$

Due to ${\phi _{12}}(t) \ge 0$, it is shown that $\frac{1}{{{c_1}}}({\delta _2} - {\delta _3})(1+\theta (t))E \ge 1$.

The third situation is that ${\phi _{12}}(t) = 0$ in set $\left\{ {\left. t \right| {\varepsilon ^*}(t) = 0} \right\}$, then the optimal control formulae can be written as:

48 $$\begin{aligned} \begin{aligned} \ 0 = {\varepsilon ^*}(t) = \frac{1}{{{c_1}}}[({1+\theta (t))E })- {\phi _{11}}].\ \end{aligned} \end{aligned}$$

Based on the above situation, the final optimal control formulae of ${\varepsilon ^*}(t)$ can be written as ${\varepsilon ^*}(t) = \min \left\{ {1,\max \left\{ {0,\frac{{({\delta _2} - {\delta _3})(1+\theta (t))E}}{{{c_1}}}} \right\} } \right\}$. Similarly, the final optimal control formulae of ${\theta ^*}(t)$ can be written as ${\theta ^*}(t) = \min \left\{ {1,\max \left\{ {0,\frac{{({\delta _2} - {\delta _3})\varepsilon (t)}}{{{c_2}}}}\right\} }\right\}$.

So far, we get the optimal control system includes state system (29) with the initial conditions *S*(0), *E*(0), *I*(0), *R*(0) and the adjoint system (37) with boundary conditions with the optimization conditions. The optimal control system can be written as:

49 $$\begin{aligned} \begin{aligned} \ \left\{ {\begin{array}{*{20}{l}} {\frac{{dS}}{{dt}}}&{} = &{}{B - \alpha SI -\alpha SI -\mu S,}\\ {\frac{{d{E}}}{{dt}}}&{} = &{}{\alpha SI - \min \left\{ {1,\max \left\{ {0,\frac{{({\delta _2} - {\delta _3})(1+\theta (t))E}}{{{c_1}}}} \right\} } \right\} }\\ {}&&\quad- {\min \left\{ {1,\max \left\{ {0,\frac{{({\delta _2} - {\delta _3}){\varepsilon (t)}}}{{{c_2}}}} \right\} } \right\} {E} - {\omega }{E} - \mu {E},}\\ {\frac{{dI}}{{dt}}}&{} = &{}{\min \left\{ {1,\max \left\{ {0,\frac{{({\delta _2} - {\delta _3}){(1+\theta (t))E}}}{{{c_1}}}} \right\} } \right\} }\\ {}&&\quad -{\min \left\{ {1,\max \left\{ {0,\frac{{({\delta _2} - {\delta _3}){\varepsilon (t)}}}{{{c_2}}}} \right\} } \right\} {E} - {\gamma }{I} - \mu {I},}\\ {\frac{{dR}}{{dt}}}&{} = &{}{\gamma I + {\omega }{E} - \mu R,}\\ {\frac{{d{\delta _1}}}{{dt}}}&{} = &{}{({\delta _1} \!-\! {\delta _2})\alpha I +\! {\delta _1}\mu ,}\\ {\frac{{d{\delta _2}}}{{dt}}}&{} = &{}{1+({\delta _2} - {\delta _3}) \varepsilon (t)(1+\theta (t))+({\delta _2} - {\delta _4})\omega -\delta _2 \mu ,}\\ {\frac{{d{\delta _3}}}{{dt}}}&{} = &{}{1 + ({\delta _1} - {\delta _2})\alpha S +\delta _3(\gamma +\mu ) -\delta _4\gamma },\\ {\frac{{d{\delta _4}}}{{dt}}}&{} = &{}{{\delta _4}\mu ,} \end{array}} \right. \ \end{aligned} \end{aligned}$$

and

50 $$\begin{aligned}{} & {} \begin{aligned} \ S(0) = {S_0},{E}(0) = {E_{0}},I(0) = {I_0},R(0) = {R_0},\ \end{aligned} \end{aligned}$$

51 $$\begin{aligned}{} & {} \begin{aligned} \ {\delta _1}({t_f}) = {\delta _2}({t_f}) = {\delta _3}({t_f}) = {\delta _4}({t_f}) = 0.\ \end{aligned} \end{aligned}$$

## Numerical simulations

The diffusion of knowledge within the digital regional innovation ecosystem has dynamic and abstract characteristics. Therefore, in order to more intuitively reflect the dynamic changes in knowledge diffusion within the innovation ecosystem^49^ . In this section, some numerical simulations will be conducted using the Runge Kutta algorithm. The numerical simulation results demonstrate the rationality of the theory. Due to the lack of a clear range of parameters in previous studies. Therefore, this article combines the basic regeneration number $R_0$ The value and stability conditions of are given in the reference^50^ , which provides the numerical values of the parameters in the model.

In order to verify the locally and globally asymptotically stability of knowledge-free equilibrium in Theorems 1 and 2. Let $B = 1,\alpha = 0.010,\varepsilon = 0.001,$

**Figure 3:**
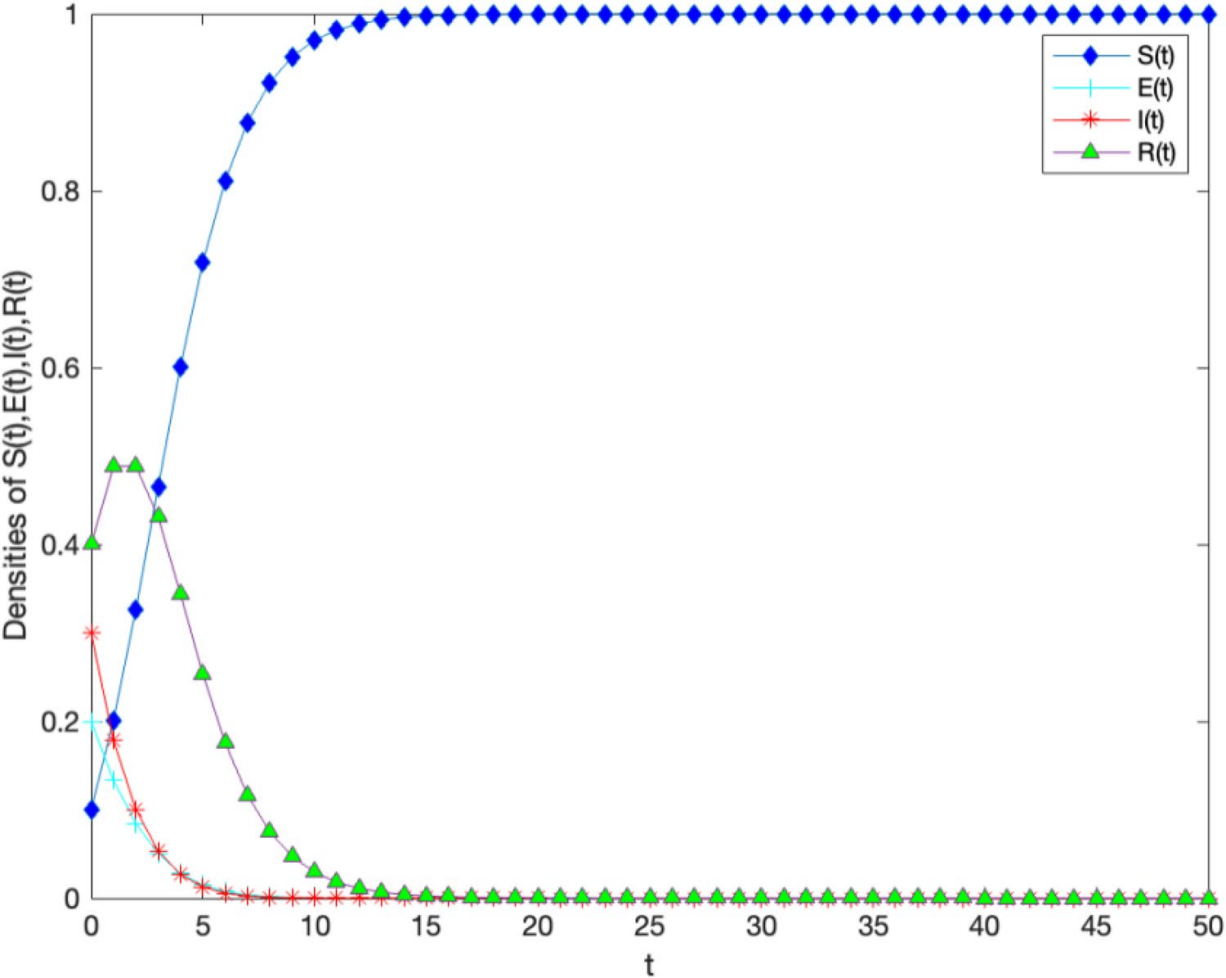
The stability of information-free equilibrium $E^0$ of system 1 with $R_0<1$.

$\theta =0.300,\mu = 0.500,{\gamma } = 0.400,\omega = 0.300$. Through calculation, it can be concluded that $R_0=0.0060<1$. Figure 3 verifies the stability of the model, indicating that over time, the number of knowledge holders shows an increasing trend in the early stages of the digital regional innovation ecosystem, with a significant increase until it tends towards 1, at this point, the digital regional innovation ecosystem has reached a stable state.In the growth stage of regional innovation ecosystem under digitization, the number of potential knowledge diffusion subjects gradually decreases until it tends to 0. In the mature stage of the regional innovation ecosystem under digitization, the number of knowledge diffusion subjects begins to decrease slightly, and then shows a clear downward trend. The knowledge diffusion subjects and knowledge immune subjects eventually converge to 0. In the end, only the knowledge immune agent remains in the system and reaches a stable state.

**Figure 4:**
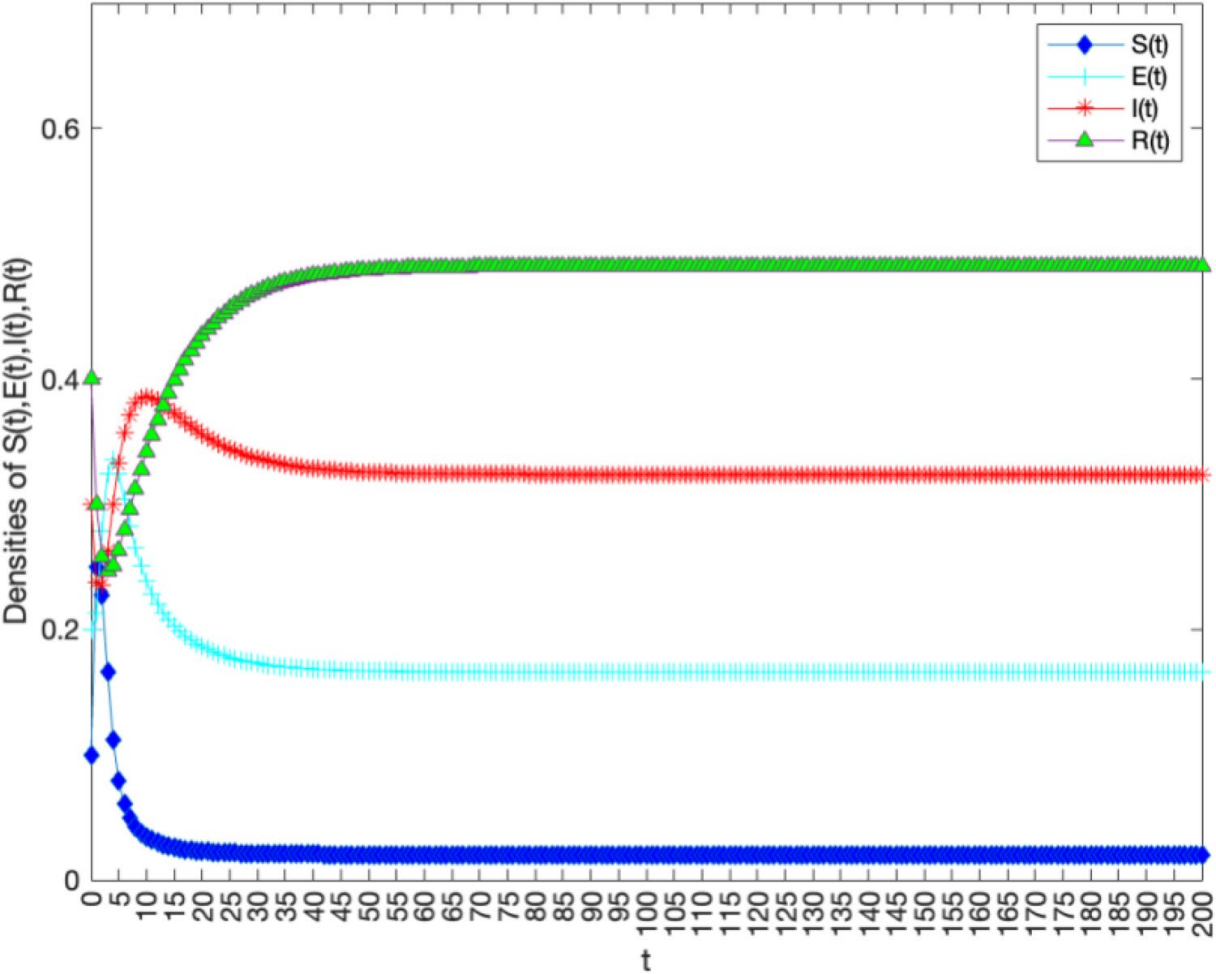
The stability of knowledge-existence equilibrium $E^*$ of system 1 with $R_0>1$.

In order to verify the locally and globally asymptotically stability of knowledge-existence equilibrium in Theorems 3 and 4. Let $B = 5,\alpha = 0.300,\varepsilon = 0.300,\theta =0.300,\mu = 0.100,{\gamma } = 0.100,\omega = 0.100$. Through calculation, it can be concluded that $R_0=7.041>1$. Figure 4 verifies the stability of the model, indicating that over time, in the early stages of the digital regional innovation ecosystem, the number of knowledge holders rapidly decreases in a short period of time, while potential knowledge diffusion subjects begin to significantly increase.In the growth stage of the regional innovation ecosystem under digitization, the number of potential knowledge diffusion subjects decreases while the number of knowledge diffusion subjets eventually stabilizes after a small decrease. In the mature stage of the digital regional innovation ecosystem, the knowledge holders, potential knowledge subjects, knowledge diffusion subjects, and knowledge immune subjects within the digital regional innovation ecosystem eventually converge to $E^*$.

In order to reveal the influence of optimal control pair $({\varepsilon ^*},{\theta ^*})$ on variety groups when we adopt the optimal control strategy. We give the image of “optimal control ($\varepsilon =\varepsilon ^*(t),\theta =\theta ^*(t)$)”, “middle control measure”, “single control measure” and “constant control measure” respectively.

**Figure 5:**
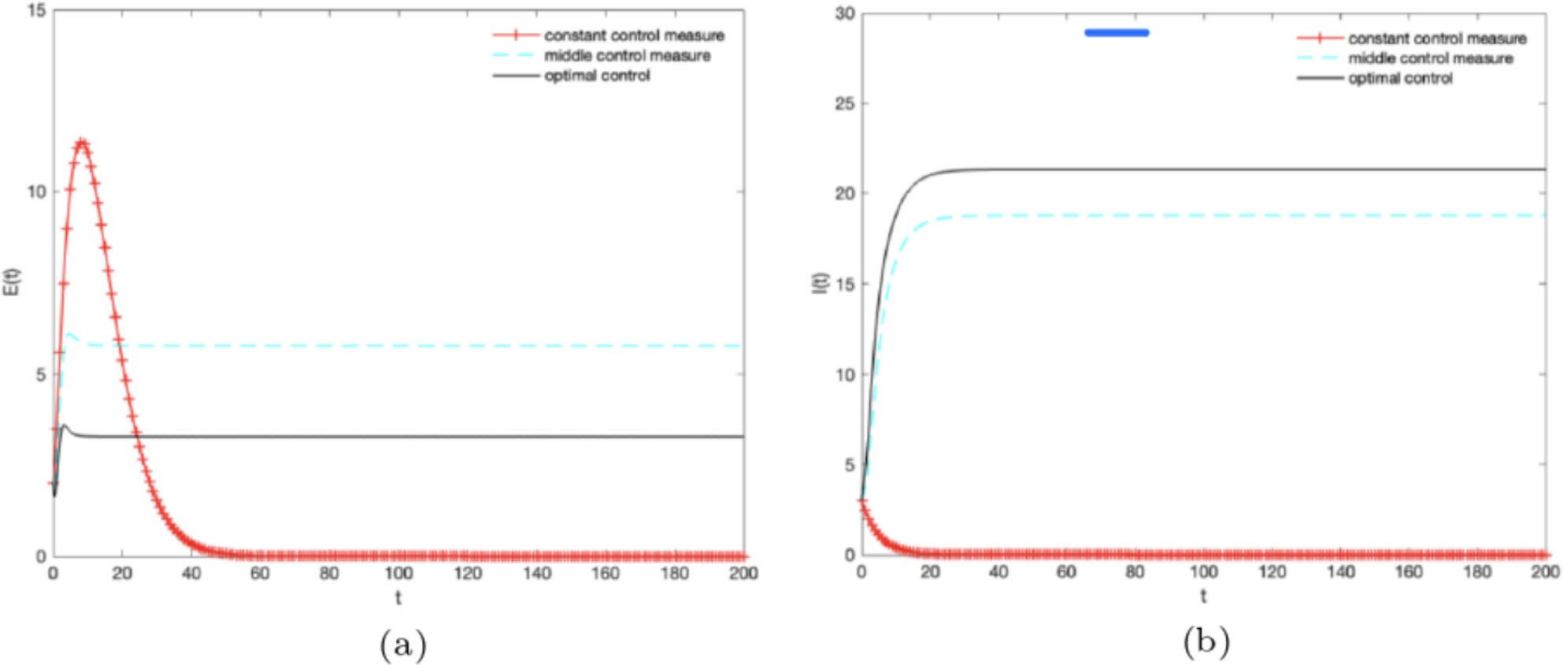
The densities of (**a**) ${E}(t),\,(\textbf{b}) {I}(t)$ change over time under different control strategies, where $B = 5,\alpha = 0.3,\omega = 0.1,{\gamma } = 0.1,\mu =0.1,\varepsilon (t) = 0.0.5.1,\theta (t) = 0.3$.

Firstly, we aim to increase the proportion of knowledge diffusion subjects and adopt different control strategies. Let $B = 5,\alpha =0.3,\omega =0.1,\gamma =0.1,\mu =0.1,\theta ^*(t)=0.3,\varepsilon (t)=0,0.5,1$ and different control strategies are adopted at the same time. Figure 5a,b illustrate the densities of *E*(*t*), *I*(*t*) change over time under different control strategies. The densities of *E*(*t*), *I*(*t*) show that “optimal control” is better than “middle control measure” batter than “single control measure” batter than “constant control measure” in Fig. 5a,b. It illustrates that the knowledge is effectively extended when the optimal control strategy is adopted. However, the density of *E*(*t*) should be negative correlation with information extension in the theoretical analysis. It is inconsistent with the phenomenon shown in Fig. 5a. Therefore, the further analysis is needed.

**Figure 6:**
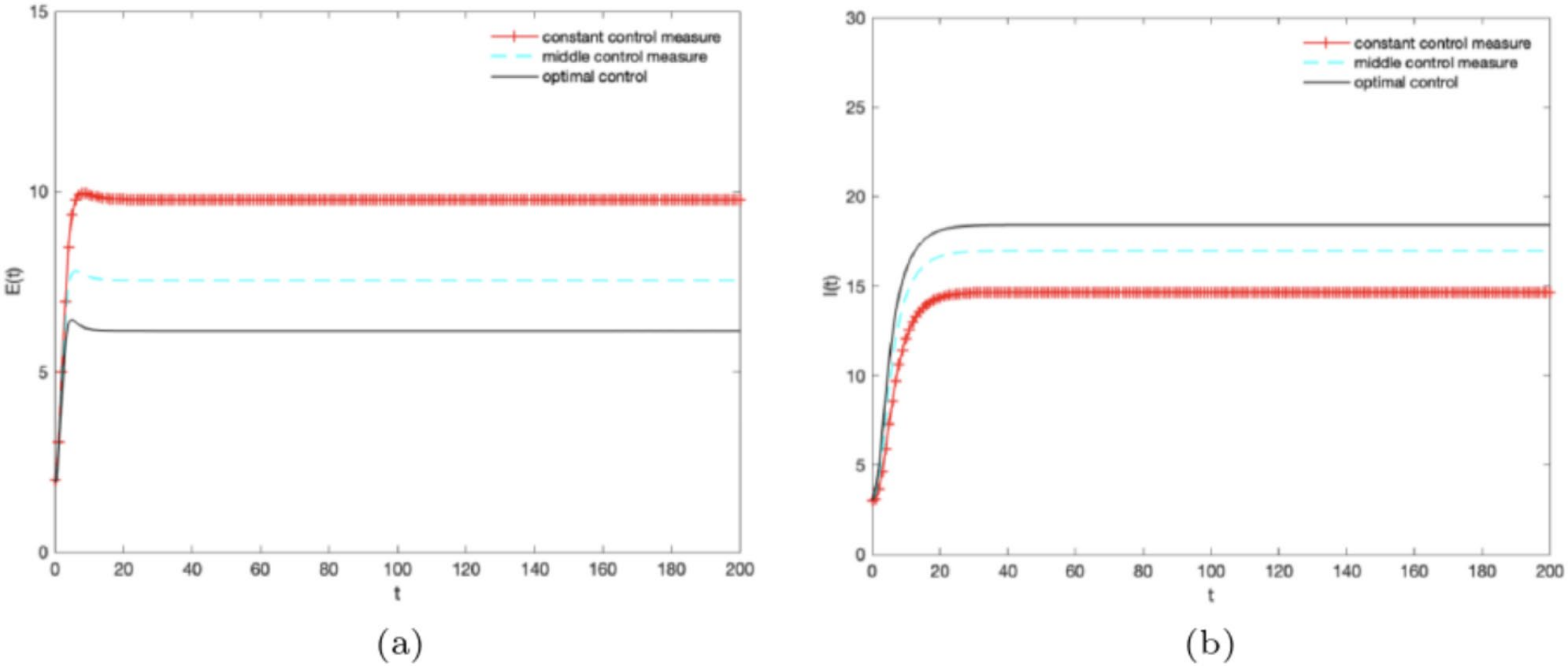
The densities of (**a**) ${E_1}(t),\, (\textbf{b}){I}(t)$ change over time under different control strategies, where $B = 5,\alpha = 0.3,\omega = 0.1,{\gamma } = 0.1,\mu =0.1,\varepsilon (t) = 0.3,\theta (t) = 0,0.5,1$.

Then, let $B = 5,\alpha = 0.3,\omega =0.1,\gamma =0.1,\mu =0.2,\varepsilon (t) = 0.3,\theta (t) = 0,0.5,1$ and different control strategies are adopted at the same time. Figure 6a,b illustrate the densities of *E*(*t*), *I*(*t*) change over time under different control strategies. The density of ${E_1}(t)$ can achieve the optimal state when “middle control measure” is adopted shown in Fig. 6a. The density of ${E_2}(t)$ is less than the “middle control measure” and “single control measure $\beta$” when optimal control strategy is adopted shown in Fig. 6b. It illustrates that the density of ${E_2}(t)$ is gradually showed a negative correlation with information extension with the increase of *B*. As shown in Fig. 6a , when the “optimal control measure” is adopted, the number (proportion) of potential knowledge subjects *E*(*t*) reaches the optimal state. As shown in Fig. 6b , as the values of control parameters continue to increase, the ratio of knowledge diffusion subject *I*(*t*) gradually shows a positive correlation with knowledge diffusion when using the “optimal control measure”, and the ratio of I (t) can reach the optimal state.

**Figure 7:**
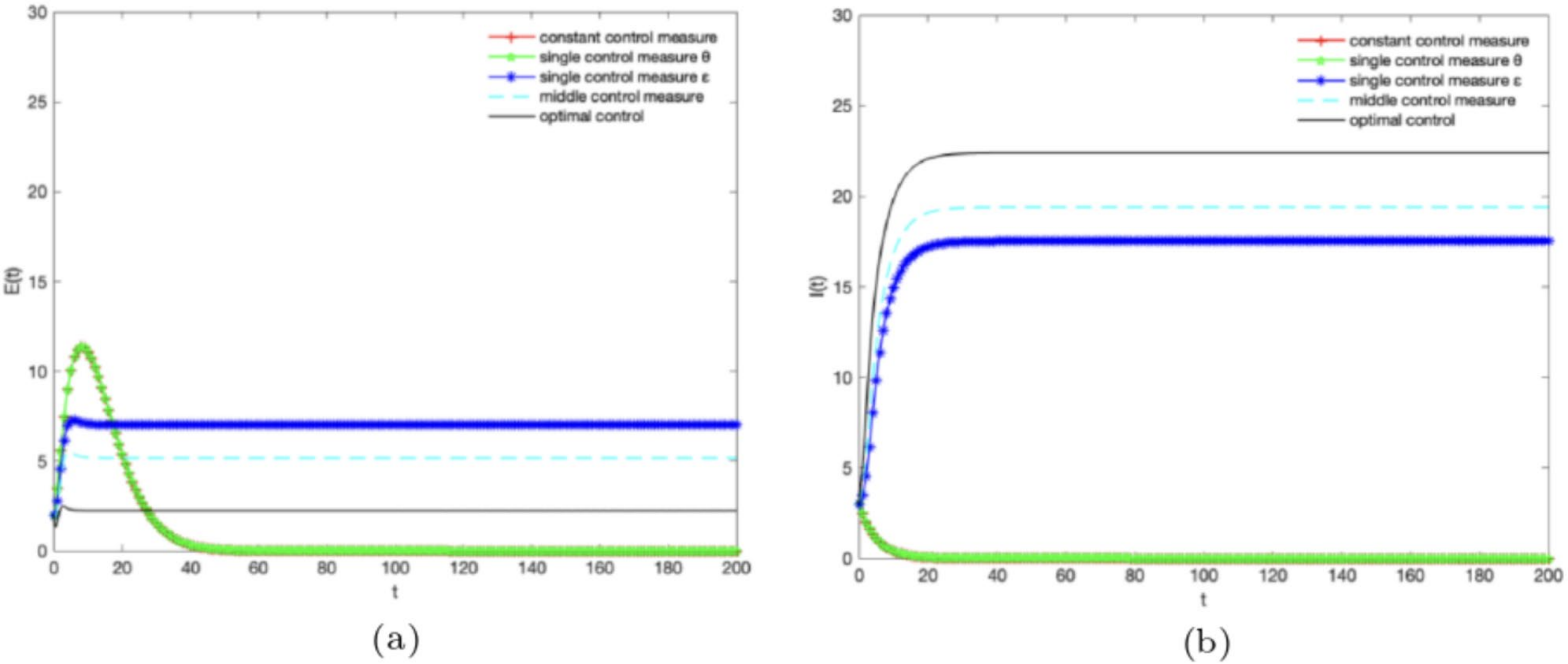
The densities of (**a**) ${E}(t),\,(\textbf{b})I(t)$ change over time under different control strategies.

Finally, let $B = 5,\alpha = 0.3,\omega =0.1,\gamma =0.1,\mu =0.1,\varepsilon (t) = \theta (t) = 0,0.5,1$ and different control strategies are adopted at the same time. Figure 7a,b show the variation of the number of *E*(*t*) and *I*(*t*) over time under different control strategies.From the ratios of *E*(*t*) and *I*(*t*) in Fig. 7, it can be seen that the “optimal control measure” is superior to the “middle control measure” and superior to the “single control measure” and superior to the “constant control measure”.As shown in Fig. 7a, when the optimal control strategy is adopted, knowledge is effectively diffused and the ratio of *E*(*t*) begins to decrease, which is negatively correlated with knowledge diffusion.As shown in Fig. 7b, when the system adopts optimal control, the ratio of *I*(*t*) will gradually increase over time, which can promote the optimal diffusion of knowledge.

Based on the above analysis, when the system adopts the optimal control strategy, the agent ratio of knowledge diffusion reaches the optimal state. Meanwhile, the influence of the density of*E*(*t*) on knowledge diffusion decreases gradually when taking the optimal control strategy with the increase of *B*.These phenomena demonstrate that the knowledge has been effectively diffused when the optimal control strategy is adopted for optimal control pair $({\varepsilon ^*},{\theta ^*})$.

## Sensitivity analysis

To discuss the effect of control variables $\varepsilon$ and $\theta$ on the basic reproductive number $R_0$, we need to perform the sensitivity analysis of $R_0$. According to the deduction above, we can figure out ${R_0} = \rho (F{V^ {-1} }) = \sqrt{\frac{{B\alpha \varepsilon (1 + \theta )}}{{\mu [ \varepsilon (1+\theta )+\omega +\mu ](\gamma +\mu ) }}}$, thereby calculating:

52 $$\begin{aligned}{} & {} \begin{aligned} \ \frac{{\partial {R_0}}}{{\partial \varepsilon }} = {\frac{{B \alpha (\omega + \mu )(1 + \theta )}}{{ 2\mu (\gamma +\mu )[\varepsilon (1+\theta )+\omega +\mu ^2]\sqrt{\frac{B\alpha \varepsilon (1+\theta )}{\mu (\gamma +\mu )[\varepsilon (1+\theta )+\omega +\mu ]}} }}} > 0,\ \end{aligned} \end{aligned}$$

53 $$\begin{aligned}{} & {} \begin{aligned} \ \frac{{\partial {R_0}}}{{\partial \theta }} = {\frac{{B \alpha (\omega + \mu )}}{{ 2\mu (\gamma +\mu )(\varepsilon \theta +\omega +\mu +\varepsilon )^2\sqrt{\frac{B\alpha \varepsilon (1+\theta )}{\mu (\gamma +\mu ) [\varepsilon (1+\theta )+\omega +\mu ]}} }}} > 0,\ \end{aligned} \end{aligned}$$

As can be seen, $R_0$ increases along with $\varepsilon$. This indicates that the knowledge diffusion subjects in the digital regional innovation ecosystem can promote knowledge diffusion.In other words, the larger the proportion of knowledge diffusion subjects, the wider the scope of knowledge diffusion.Meanwhile, $R_0$ is also positively correlated with $\theta$. This indicates that the more diverse digital platforms and channels are available for potential knowledge diffusion entities, the higher the probability of their transformation into knowledge diffusion subjects, and the wider the scope of knowledge diffusion.

**Figure 8:**
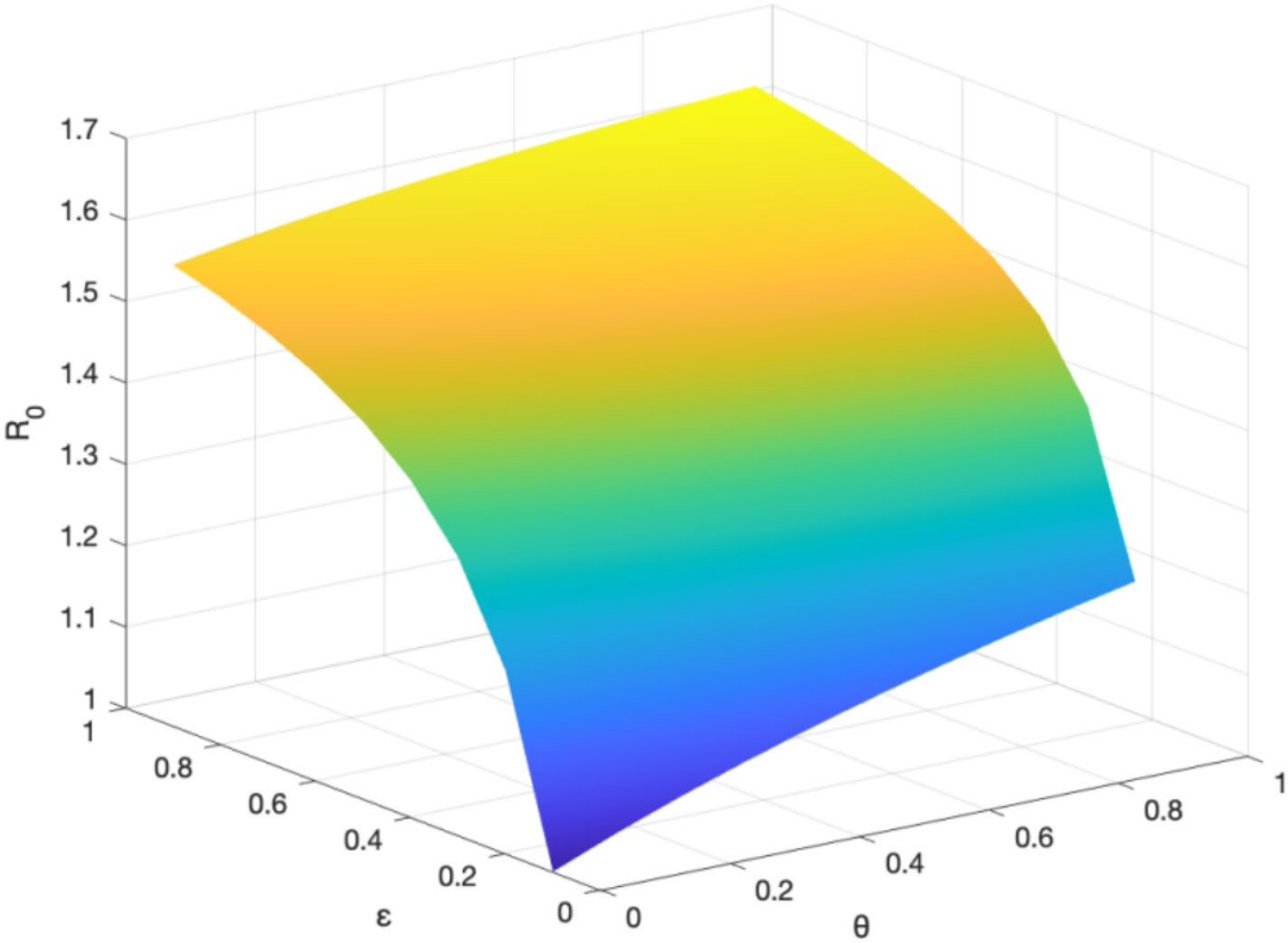
The sensitivity analysis of the basic reproduction number $R_0$.

The sensitivity analysis of $R_0$ is shown in Fig. 8.

## Research conclusions and management implications

### Research conclusions

An epidemic model is adopted in this work to clarify the mechanism of knowledge diffusion in regional innovation ecosystems under digitization. First, an SEIR model that includes potential knowledge dissemination subjects and knowledge diffusion subjects is built for coupling multiple elements during the process of knowledge diffusion in regional innovation ecosystems. This model reflects the connections and interactions between innovation agents in terms of innovative knowledge in regional innovation ecosystems under digitization, as well as the underlying evolutionary mechanism. Second, theoretical analysis is conducted on the equilibrium point and basic reproduction number of the model. It is further proven that when is less than 1, the equilibrium point without knowledge diffusion is asymptotically stable locally. On the other hand, when is greater than 1, the equilibrium point with knowledge diffusion is asymptotically stable locally. Based on this, the existence of an optimal control for the system model is validated. Afterwards, an optimal control strategy that promotes knowledge diffusion in regional innovation ecosystems under digitization is proposed and its effectiveness is validated through numerical simulations. Finally, the sensitivity of the optimal control parameters is analyzed. The following conclusions are drawn based on the aforementioned research.

First, in a regional innovation ecosystem under digitization, in order to maximize the scale and breadth of the diffusion of innovative knowledge, the knowledge-diffusion ability of innovation agents exerts a significant effect on knowledge diffusion in the system. In this work, we have quantitatively analyzed and compared the concepts of potential knowledge diffusion and knowledge diffusion from the perspective of knowledge diffusion. Unlike the traditional research regarding knowledge diffusion, the proposed optimal control strategy is based on the optimal values of the control variables.

Second, the contact rate between innovation agents affects the efficiency of knowledge diffusion in the system and the structure of the system. Therefore, increasing the scale of knowledge diffusion subjects considerably promotes the knowledge diffusion in regional innovation ecosystems under digitization. By improving their learning, transmission, and conversion of knowledge, potential knowledge diffusion subjects can become true knowledge diffusion subjects. Thus, increasing the proportion of knowledge diffusion subjects in regional innovation ecosystems under digitization accelerates knowledge diffusion, thereby increasing the efficiency of knowledge diffusion.

Third, the digital transmission ability of innovation agents affects the breadth of knowledge diffusion in the system and the self-learning ability of innovation agents affects the efficiency of knowledge diffusion in the system. In regional innovation ecosystems under digitization, the knowledge learning ability of innovation agents guarantees their acceptance and diffusion of knowledge. When innovation agents? ability to learn and receive knowledge is improved, a larger proportion of potential knowledge diffusion subjects become true knowledge diffusion subjects.

### Management implications

1. Increase the number of subjects with knowledge-diffusion ability in regional innovation ecosystems under digitization. With the continuous deepening of digitalization processes in regional innovation ecosystems, the innovation agents have formed an interactive mode based on incentive investments, such as the transformation and sharing of scientific and technological achievements. Based on this, the core enterprises establish increasingly close cooperative relationships with universities, thereby increasing the innovative knowledge holding ratio of innovation agents in regional innovation ecosystems. Supported by the digital technology *R& D* capabilities and comprehensive advantages of universities and research institutions, as well as digital technologies, such as big data, artificial intelligence, and 5G networks, regional core enterprises join hands with heterogeneous innovation agents to establish scientific and technological innovation service platforms and centers, and to achieve the integration and diffusion of high-quality innovation resources under the action of digital empowerment. The core enterprises in a system play a major role in decision-making, and conduct demand perception, innovation resources integration, and collaborative innovation based on market demands. At this point, innovation agents not only expand the channels of knowledge diffusion, but also improve the knowledge-diffusion ability, thereby increasing the infection rate of knowledge diffusion and promoting the sustainable development of regional innovation ecosystems under digitization.
2. Enhance the linkage of knowledge resources between innovation agents in regional innovation ecosystems under digitization. With the explosive growth of innovation agents and knowledge resources, the resources matching efficiency of the system have been optimized under the drive of digital technologies. In regional innovation ecosystems, the government accelerates the interactions between innovation elements by collaborating with innovation agents (such as core enterprises, universities, and research institutions) to build open laboratories, share digital platforms, etc. A novel digital regional innovation ecosystem redefines the roles of industry, academia, and research and the relationships between subjects. In addition, with the aid of the market environment, it forms a value creation model with stronger connections between innovation agents and more diffusion of knowledge resources. By optimizing the linkage of knowledge resources in a regional innovation ecosystem, we can effectively increase the conversion rate of knowledge diffusion subjects and the efficiency of knowledge diffusion in the system, thereby boosting the regional innovation efficiency.
3. Enhance the ability of innovation agents to absorb and learn the knowledge in regional innovation ecosystems under digitization. The digital technologies help heterogeneous innovation agents in regional innovation ecosystems to break down the knowledge silos. Aided by the standardized data interfaces and knowledge-sharing platforms, they rapidly integrate all kinds of explicit and implicit knowledge from various organizational spaces, modules, and departments, consequently enhancing the ability of innovation agents to absorb and learn knowledge. The digital technologies also support the construction of market demand-oriented knowledge-sharing platforms (such as Haier Makers-Lab and Xiaomi Open-Source Hardware Club). The platform subjects construct a diversified knowledge network based on specific product goals, service goals, and technical goals, and perform corresponding knowledge integration and diffusion to achieve the application and transformation of the scientific and technological achievements in different innovative technologies. In this way, the digital technologies enhance the ability of innovation agents to absorb and learn knowledge in regional innovation ecosystems under digitization, thereby increasing the infection rate of knowledge diffusion in such systems.

### Future outlook

Although this study has achieved certain research results, there are still certain problems and shortcomings. During the simulation process, no deterministic regions were selected for simulation analysis, which has certain limitations. Whether this conclusion can be promoted and applied still requires further verification of the reality of knowledge diffusion among digital manufacturing enterprises in the regional innovation ecosystem under digitization. Our future research will focus on the following three aspects. Firstly, we will consider the dissemination and diffusion of various innovative technologies and knowledge in the regional innovation ecosystem, in order to explore the different diffusion paths and mechanisms of different types of innovation resources. Secondly, when studying the knowledge diffusion of regional innovation ecosystems under digitization, empirical analysis can be introduced to enrich simulation and enhance the persuasiveness of theoretical research. Thirdly, further explore the interference mechanism of diversified knowledge diffusion in the regional innovation ecosystem under digitization, in order to achieve a more stable path for knowledge diffusion within the system.

## Data Availability

All raw data are within the manuscript.
